# Evidence for long-term potentiation in phospholipid membranes

**DOI:** 10.1073/pnas.2212195119

**Published:** 2022-12-05

**Authors:** Haden L. Scott, Dima Bolmatov, Peter T. Podar, Zening Liu, Jacob J. Kinnun, Benjamin Doughty, Ralph Lydic, Robert L. Sacci, C. Patrick Collier, John Katsaras

**Affiliations:** ^a^Neutron Scattering Division, Oak Ridge National Laboratory, Oak Ridge, TN 37831; ^b^Department of Physics and Astronomy, The University of Tennessee, Knoxville, TN 37996; ^c^Shull Wollan Center, Oak Ridge National Laboratory, Oak Ridge, TN 37831; ^d^Vanderbilt University, Nashville, TN 37235; ^e^Center for Nanophase Materials Sciences, Oak Ridge National Laboratory, Oak Ridge, TN 37831; ^f^Chemical Sciences Division, Oak Ridge National Laboratory, Oak Ridge, TN 37831; ^g^Department of Psychology, University of Tennessee, Knoxville, TN 37996

**Keywords:** lipid bilayers, droplet interface bilayers, long-term potentiation, neuromorphic, plasticity

## Abstract

Biological supramolecular assemblies, such as phospholipid bilayer membranes, have been used to demonstrate signal processing via short-term synaptic plasticity (STP) in the form of paired pulse facilitation and depression, emulating the brain’s efficiency and flexible cognitive capabilities. However, STP memory in lipid bilayers is volatile and cannot be stored or accessed over relevant periods of time, a key requirement for learning. Using droplet interface bilayers (DIBs) composed of lipids, water and hexadecane, and an electrical stimulation training protocol featuring repetitive sinusoidal voltage cycling, we show that DIBs displaying memcapacitive properties can also exhibit persistent synaptic plasticity in the form of long-term potentiation (LTP) associated with capacitive energy storage in the phospholipid bilayer. The time scales for the physical changes associated with the LTP range between minutes and hours, and are substantially longer than previous STP studies, where stored energy dissipated after only a few seconds. STP behavior is the result of reversible changes in bilayer area and thickness. On the other hand, LTP is the result of additional molecular and structural changes to the zwitterionic lipid headgroups and the dielectric properties of the lipid bilayer that result from the buildup of an increasingly asymmetric charge distribution at the bilayer interfaces.

## Significance

Lipid bilayers have the potential to be developed into neuromorphic platforms which exhibit persistent synaptic plasticity in the form of long-term potentiation (LTP), a feature associated with learning and memory. The results presented herein show that, even in the absence of peptides or proteins, lipid bilayers are capable of LTP emulating hippocampal LTP formation observed in mammals and birds. The data thus support the interpretation that the lipid bilayer provides a model for understanding the molecular basis of biological memory, as a therapeutic target for brain diseases that do not respond to drugs targeting proteins, and as a platform for artificial neural network developments and memcomputing using crossbar architectures of two-terminal passive circuit elements.

In neuroscience, synaptic plasticity—the ability to create new synapses and alter synapse density over time in response to external stimuli—plays an important role in learning and memory. This is as a result of its ability to modify synaptic strength through mechanisms that include long-term potentiation (LTP), an important mechanism for learning and memory in mammals and birds (1). Specifically, repetitive stimulation (i.e., tetanus) of hippocampal excitatory synapses, at both high (2) and low frequencies (3), produces a long-lasting increase in the strength of these synapses that persists over hours. LTP has three important properties, namely: i) cooperativity—the need to stimulate multiple afferent fibers to induce LTP; ii) input specificity—only activated signals contribute to potentiation; and iii) associativity—different inputs converge to increase their signal strength (4). Since its discovery in 1973 (1), LTP has been extensively used as a cellular model for learning and memory and has redefined the field of neuroscience (5). However, neuroplasticity and memory encoding by phospholipid membranes has yet to be seriously explored, even though lipids constitute at least 50% of the brain’s dry weight (6).

**Fig. 1.**
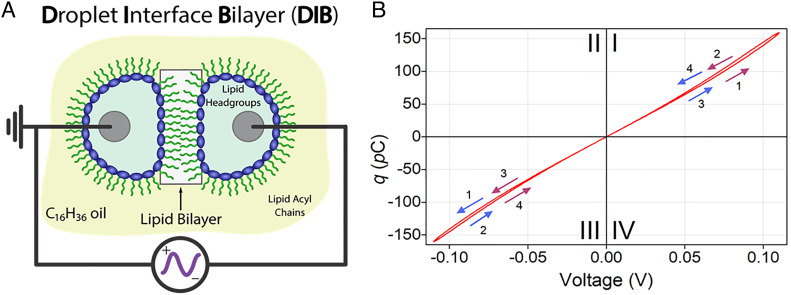
Memcapacitance and DIBs. (*A*) Schematic of a DIB made from DPhPC, hexadecane (C_16_H_34_) and water droplets. The lipid bilayer (boxed region) spontaneously forms when two lipid-coated water droplets suspended in hexadecane from silver/silver chloride (Ag/AgCl) electrodes (black lines and gray circles) are brought into close contact. As the bilayer is being formed, the hexadecane in which the droplets reside is depleted in the bilayer region due to entropic interactions (20–23). The water droplets are fractions of millimeters in diameter, while the lipid bilayer is ~4 nm thick. (*B*) Charge (the time integral of the current) versus voltage (*q-V*) in response to a sinusoidal voltage waveform results in a pinched hysteresis loop, characteristic of a memcapacitive system (20, 22). The numbered arrows indicate the directionality of the voltage polarity, from positive to negative (purple arrows) or negative to positive (blue arrows). The frequency of the sinusoidal voltage waveform is set to 0.01 Hz and varied in equal steps of 0.97 µV as follows: 0, 0.12, 0, −0.12, and 0 V (purple arrows) or 0, −0.12, 0, 0.12, and 0 V (blue arrows). It takes 99 s to collect a complete pinched hysteresis loop. Zones I and III correspond to the positive and negative pinched hysteresis lobes, with Zone I integrated areas plotted in Fig. 2*C* (*SI Appendix*, Fig. S2).

The molecular mechanisms that govern LTP in vivo are currently under debate, but it is generally accepted that membrane trafficking in postsynaptic cells is essential (7). Both presynaptic and postsynaptic sites contain molecular machinery that link the two membranes and enable them to carry out the signaling process. To address the role of the membrane in LTP, we have used droplet interface bilayers (DIBs) (8–10). Briefly, when two lipid-coated water droplets residing in a hydrocarbon solvent are brought into close contact, they can spontaneously form a planar lipid bilayer (Fig. 1*A*) which enables electrical measurements (11–13). In addition, DIBs have also been used to construct asymmetric bilayers (11, 14), complex droplet networks (15, 16), and bilayers with integral peptides and proteins (17, 18). However, central to the work described herein, is the recent study by Najem et al. that demonstrated that lipid-only DIBs are capable of memcapacitor behavior (19).

Memcapacitors, two-terminal neuromorphic circuit elements whose charge depends on their prior history and consist of different combinations of nonlinear electrical and/or ionic responses. Of these, biological supramolecular assemblies, including phospholipid bilayer membranes, offer the potential to better understand some of the underlying principles that make learning and memory in the human brain possible. Specifically, memelements with nonlinear electrical behavior—as first described by Chua (20)—offer the possibility of colocating memory and signal processing to produce biological neuronal models emulating the brain’s efficiency and flexible cognitive capabilities (21, 24, 25). Importantly, low-dimensionality soft nanomaterials such as lipid bilayers are proving to be better mimics of biological neurons and synapses than their solid-state counterparts (8, 19).

Using DIBs made from 1,2-diphytanoyl-*sn*-glycero-3-phosphocholine (DPhPC)—lipids found in Archaea extremophiles—water and hexadecane, Najem et al. showed evidence of short-term synaptic plasticity (STP) associated with memcapacitance, which was influenced not only by the frequency of the applied voltage waveform, but also the choice of hydrocarbon solvent in which the lipid-coated aqueous droplets resided (19). STP in the form of paired pulse facilitation and depression via successive voltage stimulation was ascribed to reversible changes in bilayer area and thickness (19). However, memory states associated with STP in DIB memcapacitors are volatile and cannot be stored over extended times—a key requirement for learning and memory (26).

Here, we report on the emergence of LTP in the same DIB system studied by Najem et al. (19). The difference is that here, we applied an electrical stimulation protocol for 1 hour (herein termed “training”), which consisted of a low-frequency sinusoidal voltage waveform. This electrical protocol induced a structural rearrangement of the bilayer and its headgroups, enabling LTP to manifest itself in a form that bears a striking resemblance to hippocampal LTP behavior observed in the brains of different species of animals (26–29). Importantly, in the absence of training, LTP above baseline levels was not observed, consistent with the results of Najem et al. (19). However, when LTP was present, reversal of the voltage stimulus polarity resulted in its erasure, with stored energy values returning to pretraining levels. These results bring into focus how different types of memory are stored in mammalian brains, as well as informing the next generation of neuromorphic devices.

## Results

Fig. 1*A* shows a schematic of a DPhPC DIB in hexadecane described by Najem et al. (19), where each lipid-coated water droplet was suspended from a silver/silver chloride (Ag/AgCl) electrode. When the bilayer was formed, the hexadecane in which the droplets reside in, is, for the most part, excluded from the bilayer region due to entropic depletion forces (19, 30). The droplet on one side of the membrane was electrochemically connected to the head stage electrode of a patch-clamp amplifier, while that on the other side of the membrane was connected to the ground electrode. When a sinusoidal voltage waveform of varying voltage (both positive and negative) and fixed frequency was applied across the Ag/AgCl electrodes, we observed pinched hysteresis in the plot of charge (*q*, which is the time integral of the current) versus voltage (*V*) (Fig. 1*B* and *SI Appendix*, Fig. S1*B*), the hallmark of a memcapacitive system (20–23).

**Fig. 2.**
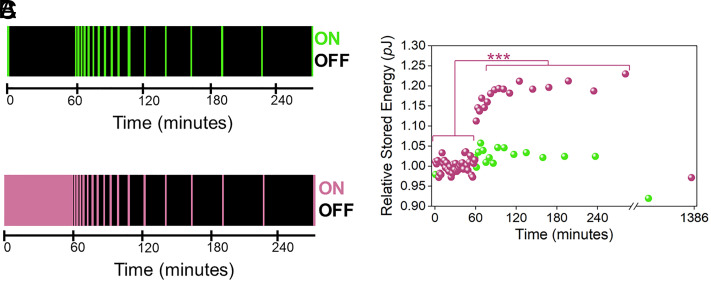
A lipid/oil/water system capable of LTP following electrical stimulation. (*A*) Temporal graphic showing that the sinusoidal voltage waveform is ON (green stripe) (Fig. 1*B*, purple arrows) at the beginning and end of the first 1-hour period (the two data points at 0 and 60 min are denoted as no training). Following this, voltage is intermittently ON in 99-s time blocks (one complete hysteresis loop), with intervening OFF periods (black stripes) increasing in duration as functions of time (see *SI Appendix* for details). (*B*) Temporal graphic showing when the sinusoidal voltage waveform is ON (purple stripes) or OFF (black stripes). The first 60 min (leftmost, solid purple stripe) denote the continuous electrical stimulation period (termed training). Following this, voltage is applied intermittently in 99-s time blocks (purple stripes), with OFF periods (black stripes) increasing in duration as functions of time (see *SI Appendix* for details). (*C*) The scaled stored energy values obtained from integrating the areas of the positive lobes of the *q-V* pinched hysteresis loops as functions of time in Zone I, for both the training (purple dots) and no training (green dots) data (see Fig. 1*B*). There is a statistically significant increase in stored energy values (purple dots) after the training period (first 60 min), whereas in the absence of training, stored energy values show no statistically significant increase (green dots). The Zone III training data are plotted in *SI Appendix*, Fig. S2*C*. For clarity, only representative training data are shown. All training data are provided in *SI Appendix*, Fig. S2 *B* and *C*. Note, that after an approximately 17-hour OFF period, the stored energy returns to baseline for the training data and decreases to a value slightly lower than the initial measurement at 0 min for the no-training data. The data have been vertically scaled so that the initial 60-min period has an average relative stored energy of 1 to allow for a direct comparison between the training and no training data. ***P ≤ 0.001.

Using the DPhPC DIB system shown in Fig. 1*A*, we applied an electrical stimulation protocol (i.e., training) —analogous to repetitive, high-frequency tetanic stimulation—consisting of a low-frequency sinusoidal voltage waveform. Fig. 2*A* shows single loops (ON, green stripes) of the low-frequency (0.01 Hz) sinusoidal voltage waveform (±0.12 V, in equal steps of 0.97 µV), applied at the onset and conclusion of a 60-min OFF period (black stripes), followed by increasingly longer OFF periods, separated by single loop ON periods. The single loops, which bookend the first 60-min OFF period, were used to establish the baseline for the membrane-stored energy. Fig. 2*B* is like Fig. 2*A*, except that the sinusoidal voltage waveform in the first 60 min was applied without interruption (training)—equivalent to tetanic stimulation at low frequencies.

When plotting stored energies (10^−12^ J or *p*J) obtained from the integrated areas of the Zone I pinched hysteresis loops (e.g., Fig. 1*B*) as functions of time, those from the untrained membrane (green dots) show no statistically significant change over 240 min, compared with the initial baseline values recorded during the first 60 min. (Fig. 2*C*). However, when the sinusoidal voltage waveform was applied continuously over the 60-min training period (Fig. 2*B*, purple stripe), the stored energy versus time plot (Fig. 2*C*, purple dots) for this bilayer was highly nonlinear and remarkably different from the untrained membrane (Fig. 2*C*, green dots) (see *SI Appendix*, Fig. S2 for Zones I and III integrated energy values). Fig. 2*C* can thus be summarized as follows: i) stored energy remains largely unaltered over the first 60-min period for both untrained (green dots) and trained bilayers (purple dots); and ii) trained bilayers exhibit LTP (i.e., an increase in membrane-stored energy), while untrained bilayers do not (stored energy is largely unaffected, also see *SI Appendix*, Fig. S2 *B* and *C*). After ~17 OFF hours, membrane-stored energy dissipated and returned to baseline values (Fig. 2*C* and *SI Appendix*, Fig. S2 *B* and *C*). Training invoking tetanic stimulation is, therefore, key to accessing LTP in lipid bilayers and is the result of a repetitive structural reconfiguration of the bilayer (see *Discussion*). We note that the minimum training period needed to produce LTP is approximately 8.3 min or the equivalent 5 sinusoidal voltage waveform cycles (*SI Appendix*, Fig. S5).

**Fig. 3.**
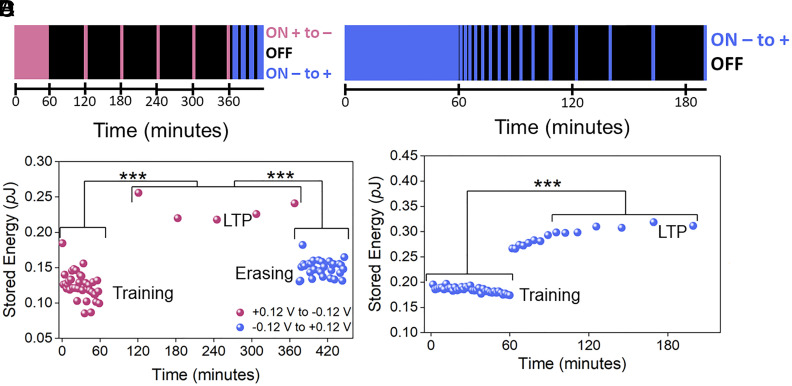
Reversing the polarity of the applied voltage waveform alters the asymmetric charge distribution at the bilayer interfaces. (*A*) Temporal graphic like Fig. 2*B*, except the blue bars correspond to negative voltage directionality (0, −0.12, 0, 0.12, and 0 V in steps of 0.97 µV). The applied voltage protocol consists of an initial 60-min training period (purple stripe) followed by a 60-min OFF period (black stripe). Subsequent 99-s ON periods (purple stripes) are followed by 60-min OFF periods (black stripes). After approximately 360 min, the direction of the sinusoidal waveform is altered (blue stripes) (see *SI Appendix* for details). (*B*) Stored energy values obtained using the applied voltage protocol in *A*. The data reveal a similar trend as those in Fig. 2*C* (purple dots) but revert toward baseline values when the direction of the voltage is reversed in a series of 10 min ON and 5 min OFF periods (blue dots). The increase in stored energy from training to LTP and the decrease in stored energy from LTP to erasing are both statistically significant. (*C*) Temporal graphic showing when the sinusoidal voltage is ON (blue) or OFF (black)—ON corresponds to the applied voltage sequence of 0, −0.12, 0, 0.12 and 0 V, in equal steps of 0.97 µV (Fig. 1*B*, blue arrows) and differs from Fig. 2*C* in that the initial applied voltage is negative, instead of positive. (*D*) Energy values obtained from the areas of the positive lobes (Fig. 1*B*, Zone I) of *q*-*V* pinched hysteresis loops as functions of time. Reversing the voltage scan direction reverses the behavior of the two lobes such that now the positive lobe displays only half the increase in stored energy over baseline compared with the positive lobe in Fig. 2*C* (purple dots). Zone III data are shown in *SI Appendix*, Fig. S3*B* and are comparable to the data in Fig. 2*C* (purple dots). Stored energy values in Fig. 3 *B* and *D* are statistically different between training and LTP. ***P ≤ 0.001.

To better understand the molecular mechanism that gives rise to LTP in a simple membrane system, we altered the way the sinusoidal voltage waveform was applied. Fig. 3*A* shows the same training protocol as in Fig. 2*C* (purple dots); however, this time it was followed by equally spaced ON and OFF signals. The LTP phase in Fig. 3*B* follows a trend similar to what is shown in Fig. 2*C* (purple dots), with two exceptions: i) in the absence of any intermediate measurements, LTP still shows an increase in stored energy of 0.12 *p*J—similar to Fig. 2*C* (purple dots); and ii) when voltage polarity is reversed and applied continuously (i.e., negative direction, Fig. 1*B*), LTP is eliminated, and the system’s stored energy returns to baseline values (Fig. 3*B*, blue dots). It should be noted that applying the voltage polarity in the reverse direction using single loops of the sinusoidal voltage waveform does not eliminate LTP (*SI Appendix*, Fig. S6). Fig. 3*C* follows the same training protocol as in Fig. 2*B*, with the exception that the pinched hysteresis loops started from negative instead of positive voltage (Fig. 1*B*). Compared with Fig. 2*C*, the increase in stored energy is less in Fig. 3*D*, but comparable with the increase observed in *SI Appendix*, Fig. S3*B* (blue dots). This shows that the starting polarity (+/−) of the sinusoidal waveform dictates whether the hysteresis lobes in Zone I or Zone III of the *q-V* plane exhibit a larger area, corresponding to increased stored energy.

## Discussion

**Fig. 4.**
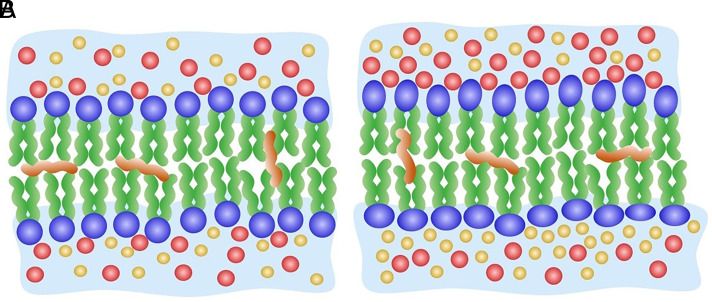
Pre- and posttrained lipid bilayers. (*A*) Schematic of a pretrained or neat lipid bilayer showing an equal distribution of K^+^ (red spheres) and Cl^−^ (yellow spheres) ions on both sides of the bilayer. (*B*) During training, using a low-frequency sinusoidal voltage waveform, K^+^ ions preferentially associate with one side of the bilayer, while the Cl^−^ counterions preferentially associate with the other side, but at lower numbers due to a drop in voltage across the highly resistive bilayer and to different affinities of the zwitterionic DPhPC headgroups for the two ions. The result is an asymmetrically charged lipid bilayer that drives the structural rearrangement of the lipids making up the bilayer, including their zwitterionic headgroups (blue spheres and ovals). Small amounts of hexadecane (orange squiggles) are shown interspersed in the hydrophobic portion of the bilayer in both (*A*) and (*B*).

Using DIBs, we show evidence of LTP in a membrane system after a sinusoidal voltage waveform is applied without interruption for 60 min. To understand this unexpected behavior, we refer to Fig. 4. Initially, K^+^ and Cl^−^ ions present in the aqueous droplets are equally distributed on both sides of the bilayer (Fig. 4*A*). However, over time, charge asymmetry of the membrane develops (Fig. 4*B*) due to energy considerations and the differential mobility of cations versus anions in the membrane (31, 32). Under positive voltage (Fig. 1*B*, Zone I), K^+^ ions preferentially partition to one side of the bilayer, while the Cl^−^ counterions partition to the other side, but at lower numbers due to a drop in voltage across the highly resistive bilayer (Fig. 4*B*). In addition, zwitterionic phosphatidylcholine headgroups have different affinities for K^+^ and Cl^−^ ions, resulting in K^+^ ions penetrating deeper into the bilayer than Cl^−^ ions (33, 34). Under negative voltage, the inverse takes place. When the sinusoidal waveform is applied continuously for 60 min—equivalent to low-frequency tetanic stimulation—the result is a progressively asymmetrically charged membrane. Altering the membrane’s permeability to K^+^ and Cl^−^ ions using shorter alkane hydrocarbons (i.e., decane or dodecane) that are incorporated in larger amounts in the membrane than hexadecane results in progressively less asymmetrically charged membranes, to the point that LTP becomes nonexistent in membranes assembled in decane (*SI Appendix*, Fig. S7) (30).

The process of an increasing asymmetric membrane can be modeled as an *RC* equivalent circuit consisting of a capacitor with progressively increasing capacitance in parallel to a high resistance. During training, the increasingly charged membrane couples to the ionic mobility in the membrane and/or intermolecular interactions at the lipid headgroup–water interface, which further amplify membrane charge asymmetry. Specifically, each current-voltage cycle progressively increases the electric field (and dipole potential) across the membrane, which in turn, increases the stored capacitive energy during training. The increased electric field orients the dipoles in the membrane, resulting in the further accumulation of K^+^ and Cl^−^ ions on either side of the bilayer. LTP is either not present in the absence of training (Fig. 2*B*, green dots) or eliminated when reversing the sinusoidal waveform polarity (Fig. 3*B*). Nevertheless, the fact that we observe LTP in a DIB membrane is surprising, as lipid molecules and lipid bilayers experience a range of dynamics over a much smaller range of time (sub ns-100 s of ns) and length (sub nm-100 s of nm) scales (35, 36), while our measurements are on the millimeter length scale and hours time scale.

We use Eq. 1 to phenomenologically describe the system. Specifically, the integration of the *q-V* hysteresis loops determines the total energy supplied to the system and can be written as follows (19):

[1] $$\int_{0}^{q}Vdq=\frac{C{\left(V\right)}^{2}}{2}-\frac{C_{0}{\left(V_{0}\right)}^{2}}{2}+\frac{1}{2}\int_{C_{0}}^{C}V^{2}dC,$$

where the first two terms correspond to LTP and the capacitive energy stored in the membrane, respectively. The third term in Eq. 1 is the energy dissipation that drives the changes in capacitance—*C_0_* is the membrane capacitance at zero membrane potential (*V_0_*). The memory terms are responsible for the inequivalence between the supply energy (equivalent to a battery) and the energy stored in the membrane. Importantly, the dissipated energy is being continuously replenished during the training period as the system is driven into a nonequilibrium steady state. This results in progressive changes to the membrane capacitance (memory terms) while the nonequilibrium steady state is maintained. The stored energy persists even after the applied voltage has been turned off and represents an energy term that depends on its history, and which manifests itself as LTP. In the case of a symmetrically charged membrane prior to training, the stored capacitive energy [C_0_(V_0_^2^) / 2] is equal to zero. However, after training, the interface becomes increasingly asymmetrically charged, creating additional potentials due to charge and dipolar gradients being developed across the membrane. As such, C_0_(V_0_^2^) / 2 energies are no longer zero but increase continuously as the charged interface becomes increasingly asymmetric.

It is interesting to note that the stored energies shown in Fig. 2*C* (green and purple dots) in the time interval between 1,300 and 1,400 min are less than those at 0 min. This can be rationalized using the dissipation term in Eq. 1. Of note, is that during the OFF intervals (after training in Fig. 2*C*), the dissipative memory term disappears, but not the stored energy, which continues to restructure the bilayer, resulting in the sharp increases in the potential energy after the training period ends. This is equivalent to capacitive energy-harvesting mechanisms used in self-powered nanogenerators that harness vibrational energy during movement (37) or in the case of neural stimulation (2), driven by energy harvesting from the movement of biological molecules in the environment via LTP. However, when the membrane experiences an extended OFF period over multiple hours, dissipation overtakes the energy supply term, leading to a further drop in the baseline-stored energy values.

The duration of the training period determines the amount of accumulated charge at the bilayer interfaces, saturating after only a brief period (*SI Appendix*, Fig. S5). In the brain, LTP (due to high-frequency (ms) tetanic stimulation) is generated biochemically, via membrane receptors, neurotransmitters, and other signaling biomolecules (38, 39), and can last anywhere from hours to days. For our single lipid component DIBs, the analogous molecular mechanism is the formation of an asymmetrically charged membrane due to the continuous, low-frequency electrical stimulation training protocol. Relaxation back to the symmetrically charged configuration takes many hours, which suggests that the bilayer remains highly insulative, and that small, thermally activated leakage currents are primarily responsible for the dissolution of bilayer charge asymmetry overtime (40).

Our current results are consistent with dynamic electrochemical impedance spectroscopy (dEIS) measurements on DPhPC DIBs, which revealed that structural reorganization drives the long-term memory behavior of lipid bilayer membranes (41). Time constant analysis of the dEIS data from equivalent *RC* circuits distinguished simple geometric changes responsible for memcapacitive behavior, such as bilayer thickening, from hexadecane migration into the hydrophobic core of the membrane during the molecular reorganization of the lipid acyl tails that results in changes to the intrinsic dielectric properties of the system (41).

In conclusion, the demonstration of LTP normally associated with learning and memory in animals, but in a simple lipid/oil/water system lacking peptides or proteins, presents us with a simple model for research aiming to understand the molecular basis of biological memory. These research topics can be explored in the future by changing the current chemical composition of the DIBs, the inclusion of transmembrane peptides, and the integration of different energy-state DIBs in artificial neural networks using crossbar nanoscale architectures.

## Materials and Methods

### Materials.

DPhPC was purchased from Avanti Polar Lipids (Alabaster) and D_2_O (99.9% D) from Cambridge Isotope Laboratories (Andover, MA). Both were used as supplied. H_2_O was produced using a High-Q water purification system (Wilmette, IL). Buffering solution was prepared using 4-morpholinepropanesulfonic acid (MOPS, >99.5%) and potassium chloride (KCl, 99.0 to 100.5%), both purchased from Sigma-Aldrich, St. Louis, MI). Solution pH or pD (~7.4 in both cases) was adjusted, respectively, using sodium hydroxide or sodium deuteroxide (Sigma-Aldrich). A 125-μm diameter silver wire (Goodfellow, Pittsburgh, PA) with a ball-end formed through heating was bleached to produce silver/silver chloride (Ag/AgCl) wires. The ball ends were then coated with 1% agarose (ThermoFisher Scientific, Waltham, MA). Electrodes were used in pairs. Clear photopolymer resin was purchased from Formlabs (Somerville, MA) and used to 3D print transparent reservoirs (“stadia”) filled with hexadecane (>99%, Sigma-Aldrich).

### Methods.

#### Vesicle Preparation and DIB Fabrication.

DPhPC multilamellar vesicles (MLVs) were prepared by hydrating dried DPhPC lipid films using 10 mM MOPS/500 mM KCl buffer, prepared in H_2_O or D_2_O at pH or pD values, respectively, of ~7.4. MLVs were sonicated for 30 min, followed by six freeze/thaw cycles (−80 °C and 40 °C). Lipid solutions were then extruded through polycarbonate membranes populated with 100-nm diameter pores (31 passes) using an Avanti Mini Extruder (Avanti Polar Lipids, Alabaster, AL), resulting in ~100-nm diameter large unilamellar vesicles (LUVs) at a lipid concentration of 2 mg/mL.

DIBs were formed by submerging agarose-coated Ag/AgCl electrodes into hexadecane-filled stadia using micro manipulators. 300-nL aqueous droplets containing LUVs were pipetted onto the ball ends of the electrodes, forming a lipid monolayer at the oil/water interface. After monolayer formation—which was visually inferred through the sagging of the droplets on the electrodes—the droplets were carefully brought together to form a bilayer (1)—note, that the DIBs reside below a 4-mm-thick layer of hexadecane, isolating them from the atmosphere and minimizing the possibility of evaporation (see *SI Appendix*, Table S3). An Evolve 512 camera (Photometrics, Tucson, AZ) mounted on a Nikon Eclipse TE300 inverted microscope (Melville, NY) was used to visualize the bilayer formation process in real time—it was also monitored via an increase in membrane capacitance.

#### Electrical Measurements.

Capacitance versus voltage (C-V) plots and charge versus voltage (q-V) plots were determined by applying a 10 Hz, 10-mV triangular wave to the electrodes with a function generator (Stanford Research Systems DS345, Sunnyvale, CA), overlaid on a 10 mHz, 110-mV sinusoidal voltage waveform (used in all experiments), using a custom script in LabView (National Instruments, Austin, TX). Electrical measurements were recorded using Clampex software from an Axon Instruments Axopatch 200B patch-clamp amplifier and an Axon Instruments Digidata 1550B low-noise analog-digital signal converter (Molecular Devices, San Jose, CA). To reduce extraneous electrical noise and physical vibration, all recordings were made with the entire DIB experimental setup enclosed in a custom-made Faraday cage that was grounded to the building and placed on an anti-vibration isolation table. All measurements were performed at room temperature (~23 °C).

### Data Analysis.

The low-frequency, high-amplitude sinusoidal waveform drove the changes in the membrane geometry and dielectric properties responsible for the memcapacitive behavior in the device (e.g., electrowetting), while the capacitance values were extracted from the square wave current responses of the lipid bilayer from the high-frequency triangular voltage waveform with a custom script in Igor (WaveMetrics, Portland, OR). The corresponding ionic charge was determined from q = CV. The stored capacitive energies associated with learning and memory were then determined from numerical integration of the areas of the pinched hysteretic q-V loops (*p*J) as functions of time (2) using the Igor script.

### Statistical Analysis.

Statistical analysis of stored energy values obtained from the integrated areas of q-V pinched hysteresis loops as a function of time was carried out to determine if the observed changes were statistically different. An independent-samples *t* test was used to determine if the initial baseline values during the training phase (average of three different H_2_O or D2O datasets) and the final baseline values of the long-term memory phase (average of three different H_2_O or D_2_O datasets) were statistically different. For Fig. 2 *B* and *C*, this was done for the single D_2_O dataset plotted to show statistical significance. Levene’s test was used to test for equality of variances within the *t* test, where a *P*-value of <0.05 indicates statistically meaningful differences between baseline training stored energy values and long-term memory values. Statistical analyses were performed using SPSSv27 software (IBM Analytics, Armond, NY).

## Supplementary Material

Appendix 01 (PDF)

## Data Availability

All study data featured are included in the article and/or *SI Appendix* and are available upon request.

## References

[1] T. V. Bliss, T. Lømo, Long-lasting potentiation of synaptic transmission in the dentate area of the anaesthetized rabbit following stimulation of the perforant path. J. Physiol. **232**, 331–356 (1973). 4727084 10.1113/jphysiol.1973.sp010273 PMC1350458

[2] T. V. Bliss, G. L. Collingridge, A synaptic model of memory: Long-term potentiation in the hippocampus. Nature **361**, 31–39 (1993). 8421494 10.1038/361031a0

[3] I. Abramets, Y. V. Kuznetsov, I. Samoi’lovich, Effects of low-frequency tetanic stimulation of the synaptic inputs on different forms of long-term potentiation in the rat hippocampus. Neurophysiology **31**, 310–315 (1999).

[4] E. R. Kandel , Principles of Neural Science (McGraw-hill, New York, 2000), **vol. 4**.

[5] R. A. Nicoll, A brief history of long-term potentiation. Neuron **93**, 281–290 (2017). 28103477 10.1016/j.neuron.2016.12.015

[6] J. A. Hamilton, C. J. Hillard, A. A. Spector, P. A. Watkins, Brain uptake and utilization of fatty acids, lipids and lipoproteins: Application to neurological disorders. J. Mol. Neurosci. **33**, 2–11 (2007). 17901539 10.1007/s12031-007-0060-1

[7] P.-M. Lledo, X. Zhang, T. C. Südhof, R. C. Malenka, R. A. Nicoll, Postsynaptic membrane fusion and long-term potentiation. Science **279**, 399–403 (1998). 9430593 10.1126/science.279.5349.399

[8] L. Tsofina, E. Liberman, A. Babakov, Production of bimolecular protein-lipid membranes in aqueous solution. Nature **212**, 681–683 (1966).

[9] K. Funakoshi, H. Suzuki, S. Takeuchi, Lipid bilayer formation by contacting monolayers in a microfluidic device for membrane protein analysis. Anal. Chem. **78**, 8169–8174 (2006). 17165804 10.1021/ac0613479

[10] M. A. Holden, D. Needham, H. Bayley, Functional bionetworks from nanoliter water droplets. J. Am. Chem. Soc. **129**, 8650–8655 (2007). 17571891 10.1021/ja072292a

[11] W. L. Hwang, M. A. Holden, S. White, H. Bayley, Electrical behavior of droplet interface bilayer networks: Experimental analysis and modeling. J. Am. Chem. Soc. **129**, 11854–11864 (2007). 17764183 10.1021/ja074071a

[12] J. L. Poulos, W. C. Nelson, T.-J. Jeon, C.-J.C. Kim, J. J. Schmidt, Electrowetting on dielectric-based microfluidics for integrated lipid bilayer formation and measurement. Appl. Phys. Lett. **95**, 013706 (2009).

[13] E. C. Freeman, A. B. Farimani, N. R. Aluru, M. K. Philen, Multiscale modeling of droplet interface bilayer membrane networks. Biomicrofluidics **9**, 064101 (2015). 26594262 10.1063/1.4935382 PMC4644148

[14] P. J. Milianta, M. Muzzio, J. Denver, G. Cawley, S. Lee, Water permeability across symmetric and asymmetric droplet interface bilayers: Interaction of cholesterol sulfate with DPhPC. Langmuir **31**, 12187–12196 (2015). 26492572 10.1021/acs.langmuir.5b02748

[15] Y. Elani, A. Gee, R. V. Law, O. Ces, Engineering multi-compartment vesicle networks. Chem. Sci. **4**, 3332–3338 (2013).

[16] M. Schindler, A. Ajdari, Droplet traffic in microfluidic networks: A simple model for understanding and designing. Phys. Rev. Lett. **100**, 044501 (2008). 18352282 10.1103/PhysRevLett.100.044501

[17] S. Haylock , Membrane protein mediated bilayer communication in networks of droplet interface bilayers. Commun. Chem. **3**, 1–8 (2020). 34113722 10.1038/s42004-020-0322-1 PMC7610947

[18] H. E. Findlay, N. J. Harris, P. J. Booth, “Integrating membrane transporter proteins into droplet interface bilayers” in Computational Design of Membrane Proteins (Springer, 2021), pp. 31–41. 10.1007/978-1-0716-1468-6_2 34302668

[19] J. S. Najem , Dynamical nonlinear memory capacitance in biomimetic membranes. Nat. Commun. **10**, 1–11 (2019). 31324794 10.1038/s41467-019-11223-8 PMC6642212

[20] L. Chua, Memristor-the missing circuit element. IEEE Trans. Circuit Theory **18**, 507–519 (1971).

[21] M. Di Ventra, Y. V. Pershin, The parallel approach. Nat. Phys. **9**, 200–202 (2013).

[22] L. Chua, If it’s pinched it’sa memristor. Semicond. Sci. Technol. **29**, 104001 (2014).

[23] M. Di Ventra, Y. V. Pershin, L. O. Chua, Circuit elements with memory: Memristors, memcapacitors, and meminductors. Proc. IEEE **97**, 1717–1724 (2009).

[24] K. M. Bresniker, S. Singhal, R. S. Williams, Adapting to thrive in a new economy of memory abundance. Computer **48**, 44–53 (2015).

[25] C. Mead, Neuromorphic electronic systems. Proc. IEEE **78**, 1629–1636 (1990).

[26] L. Ding, D. J. Perkel, Long-term potentiation in an avian basal ganglia nucleus essential for vocal learning. J. Neurosci. **24**, 488–494 (2004). 14724247 10.1523/JNEUROSCI.4358-03.2004 PMC6729982

[27] W. C. Clapp, J. P. Hamm, I. J. Kirk, T. J. Teyler, Translating long-term potentiation from animals to humans: A novel method for noninvasive assessment of cortical plasticity. Biol. Psychiatry **71**, 496–502 (2012). 21974785 10.1016/j.biopsych.2011.08.021 PMC3253317

[28] J. L. Martinez Jr., B. E. Derrick, Long-term potentiation and learning. Annu. Rev. Psychol. **47**, 173–203 (1996). 8624136 10.1146/annurev.psych.47.1.173

[29] M. J. Guimaraes Marques , Long-term potentiation decay and poor long-lasting memory process in the wild rodents Proechimys from brazil’s amazon rainforest. Front. Behav. Neurosci. **12**, 2 (2018). 29410617 10.3389/fnbeh.2018.00002 PMC5787059

[30] G. J. Taylor, G. A. Venkatesan, C. P. Collier, S. A. Sarles, Direct in situ measurement of specific capacitance, monolayer tension, and bilayer tension in a droplet interface bilayer. Soft Matter **11**, 7592–7605 (2015). 26289743 10.1039/c5sm01005e

[31] H. L. Tepper, G. A. Voth, Mechanisms of passive ion permeation through lipid bilayers: Insights from simulations. J. Phys. Chem. B **110**, 21327–21337 (2006). 17048962 10.1021/jp064192h PMC4129643

[32] M. M. Waegele, C. M. Gunathunge, J. Li, X. Li, How cations affect the electric double layer and the rates and selectivity of electrocatalytic processes. J. Chem. Phys. **151**, 160902 (2019). 31675864 10.1063/1.5124878

[33] A. A. Gurtovenko, I. Vattulainen, Effect of NaCl and KCl on phosphatidylcholine and phosphatidylethanolamine lipid membranes: Insight from atomic-scale simulations for understanding salt-induced effects in the plasma membrane. J. Phys. Chem. B **112**, 1953–1962 (2008). 18225878 10.1021/jp0750708

[34] U. M. Ferber, G. Kaggwa, S. P. Jarvis, Direct imaging of salt effects on lipid bilayer ordering at sub-molecular resolution. Eur. Biophys. J. **40**, 329–338 (2011). 21153636 10.1007/s00249-010-0650-7

[35] S. Gupta, R. Ashkar, The dynamic face of lipid membranes. Soft Matter **17**, 6910–6928 (2021). 34235519 10.1039/d1sm00646k

[36] E. Sezgin, P. Schwille, Fluorescence techniques to study lipid dynamics. Cold Spring Harb. Perspect. Biol. **3**, a009803 (2011). 21669985 10.1101/cshperspect.a009803 PMC3220360

[37] J. D. Phillips, Energy harvesting in nanosystems: Powering the next generation of the internet of things. Front. Nanotech. **3**, 633931 (2021).

[38] O. Hvalby , Specificity of protein kinase inhibitor peptides and induction of long-term potentiation. Proc. Natl. Acad. Sci. U.S.A. **91**, 4761–4765 (1994). 8197132 10.1073/pnas.91.11.4761 PMC43868

[39] K. B. Grey, B. D. Burrell, Co-induction of LTP and LTD and its regulation by protein kinases and phosphatases. J. Neurophysiol. **103**, 2737–2746 (2010). 20457859 10.1152/jn.01112.2009 PMC2867558

[40] A. Blicher, K. Wodzinska, M. Fidorra, M. Winterhalter, T. Heimburg, The temperature dependence of lipid membrane permeability, its quantized nature, and the influence of anesthetics. Biophys. J. **96**, 4581–4591 (2009). 19486680 10.1016/j.bpj.2009.01.062 PMC2711498

[41] R. L. Sacci , Disentangling memristive and memcapacitive effects in droplet interface bilayers using dynamic impedance spectroscopy. Adv. Electron. Mater. **8**, 2200121 (2022).

