# Supplementary material for: Evidence for long-term potentiation in phospholipid membranes
Source: Proc Natl Acad Sci U S A. 2022 Dec 5;119(50):e2212195119. doi: 10.1073/pnas.2212195119 (PMC9897439; doi:10.1073/pnas.2212195119)
Supplement: Supplementary file 1 — Appendix 01 (PDF) [file pnas.2212195119.sapp.pdf]

Supplementary Information for
Evidence for long-term potentiation in phospholipid membranes.

Haden L. Scott[a1], Dima Bolmatov[b,c1], Zening Liu[d], Benjamin Doughty[e], Ralph Lydic[f], Robert L. Sacci[e], C. Patrick Collier[d1], and John Katsaras[b,c,f,1]

[1]Haden L. Scott, Dima Bolmatov, C. Patrick Collier, John Katsaras.
Email: scotthl@ornl.gov; dbolmato@utk.edu; colliercp@ornl.gov; katsarasj@ornl.gov

This PDF file includes:

Supplementary text
Figures S1 to S10 (not allowed for Brief Reports)
Tables S1 to S3
SI References

Supplementary Information Text

Experimental memory and learning protocol. In Fig. 2D, an experimental protocol was used to access the long-term potentiation (LTP) phase in DPhPC DIBs. For the initial 60 min. training period, a continuous sinusoidal voltage waveform was applied. After this period, a series of increasingly longer OFF periods were implemented followed by a single 99 s ON period. The timing of the OFF periods was as follows: 0.2, 0.6, 1.1, 1.6, 2.1, 2.6, 3.2, 4.2, 4.7, 5.2, 7.6,12.7, 17.8, 22.5, 26.9, 35.9, and 45.1 min. This same protocol was used in Fig. S2. The experimental protocol was altered in Fig. 3B, where the initial 60 min. training period was followed by a series of equally spaced 60 min. OFF and 99 s ON periods. After this, the voltage direction was flipped, so that the ON periods started with a negative applied voltage (zone III, Fig. 1B). 10 min. ON periods (gray bars, Fig. S3) were followed by 5 min. OFF periods.

DPhPC DIBs stability. DPhPC DIBs were measured in both H_2O and D_2O aqueous solutions. Although little to no change was observed between these two isotopes in terms of stored energy (*p*J), they differed in stability (fig. S2A, B). For example, we were unable to collect data after a 17-h OFF period in H_2O DPhPC DIBS, unlike those prepared in D_2O (1, 2). Note, that in some cases, D_2O DPhPC DIBs were stable after three days, when experiments were terminated – they may remain viable experimental platforms for much longer times. This stability is maintained, in part, by the presence of a roughly 4 mm thick layer of hexadecane that prevents the droplets from being exposed to the environment, which would lead to evaporation. Measurements were taken on the same DPhPC DIB for up to ~65 h. Using images taken at various times during the experiment, the diameter of each drop was measured using ImageJ, showing that were minimal changes in droplet diameter (Table S3).

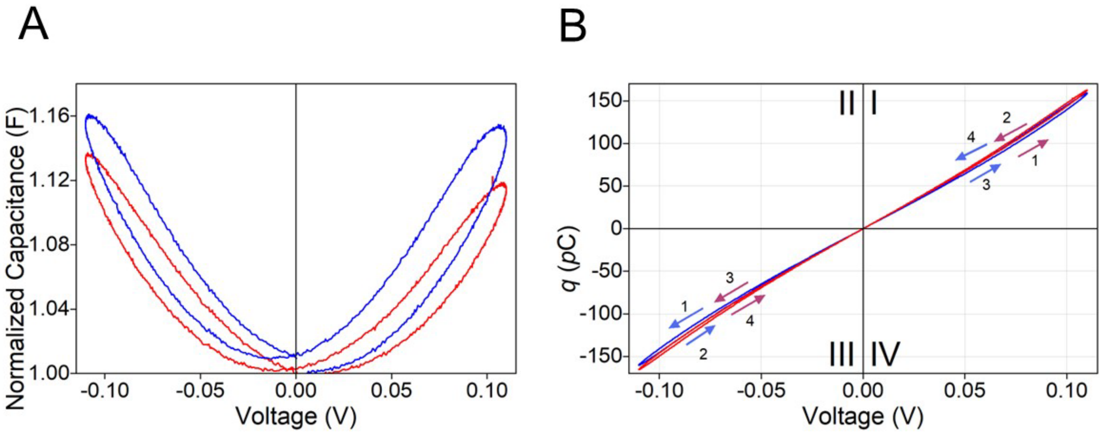

Fig. S1. Lipid bilayers display memcapacitive behavior in both H_2O and D_2O. (A) Normalized dynamical capacitance (*C*-*V*) and (B) charge versus voltage (*q*-*V*) pinched hysteresis loops of H_2O (blue) or D_2O (red) DPhPC DIBs in hexadecane, in response to a sinusoidal voltage waveform (representative data shown). (B) The numbered arrows indicate the directionality of the voltage polarity, i.e., 0, 0.12, 0, -0.12, and 0 V (purple arrows) or 0, -0.12, 0, 0.12, and 0 V (blue arrows), with a voltage frequency of 0.01 Hz.

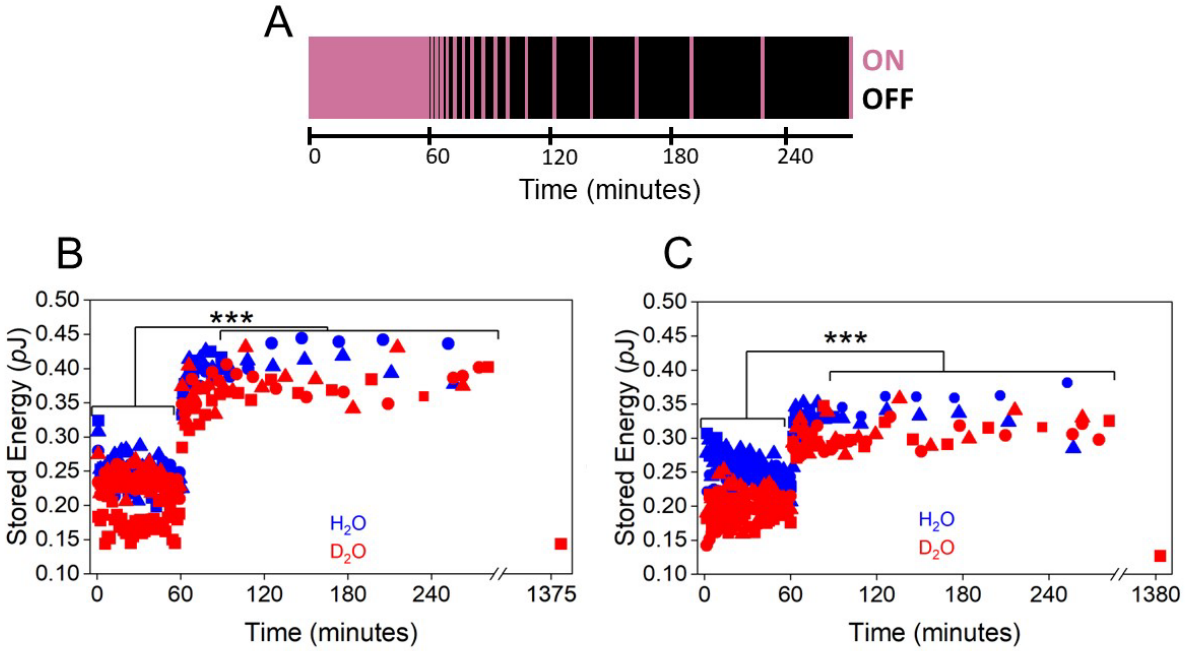

Fig. S2. DPhPC DIBs and long-term potentiation (LTP). (A) Temporal graphic showing when the sinusoidal voltage is ON (purple stripes) or OFF (black stripes) – ON corresponds to the applied voltage sequence of 0, 0.12, 0, -0.12, and 0 V, in equal steps of 0.97 μV. DIBs are trained by applying a continuous sinusoidal voltage waveform for 60 min. (leftmost, solid purple stripe). Following the 60 min. training period, the sinusoidal voltage waveform is intermittently applied in blocks of 99 s (*i.e.,* the time needed to collect 1 complete hysteresis loop), with OFF periods increasing in duration as functions of time. Except for the initial 60 min. training period, all subsequent ON events lasted for 99 s (individual purple stripes). (B-C) Stored energy values (*p*J) were obtained by integrating the area of the positive (B) or negative (C) lobe of the *q*-*V* pinched hysteresis loops (Fig. S1B, zones I and III, respectively). Red and blue symbols correspond to integrated areas from D_2O or H_2O DIBs, respectively. Note, that after a 17 h OFF period, the stored energy of a D_2O DPhPC DIB returned to baseline, however, no H_2O DPhPC DIB survived this extended OFF period. The 10 Hz, 10 mV triangle wave was always on. Average maximum values were statistically different from the average baseline training values for both D_2O and H_2O DIBs. ***$p \leq 0.001$.

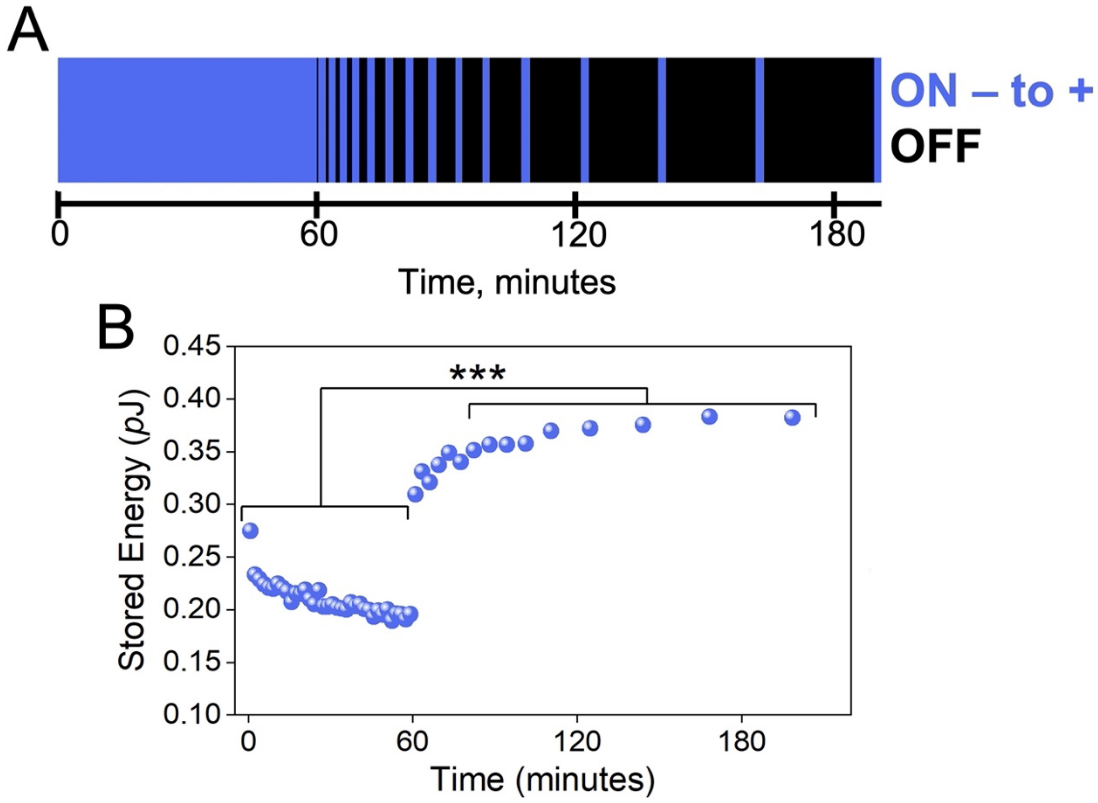

Fig. S3. **Voltage scan directionality.** (A) Temporal graphic showing when the sinusoidal voltage is ON (blue stripes) or OFF (black stripes) – ON corresponds to the applied voltage sequence of 0, -0.12, 0, 0.12, and 0 V, in equal steps of 0.97 μV (Fig. 1B, blue arrows) and differs from Fig. S2A in that the initial applied voltage has a negative polarity. (B) Energy values obtained by integrating the area of the negative *q*-V pinched hysteresis loop as a function of time in zone III (Fig. S1B). Reversing the voltage scan direction reverses the behavior of the two lobes, where now the negative lobe values display a larger change in energy, compared to the positive lobe values (Fig. 3D). This is opposite to what is observed in Fig. 2C (purple dots) and Fig. S2B. Stored energy values are statistically different between training and long-term memory. ***$p \leq 0.001$.

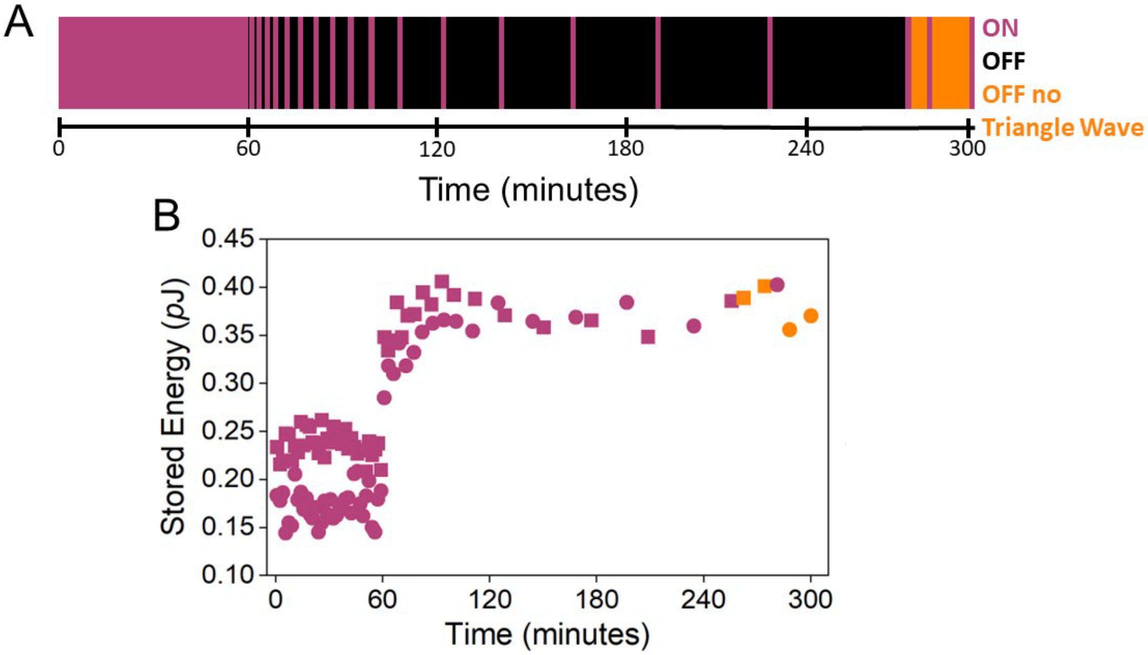

Fig S4. Triangular waveform does not affect memory. (A) Graphical representation of the sinusoidal and triangular (10 Hz, 10 mV) voltage waveforms in both ON (purple stripes) or OFF (black stripes) intervals – comparable to Fig. *S*2A. Toward the end of the applied voltage protocol, the triangular waveform is turned OFF (orange stripes) for 5 and then 10 min. (B) Stored energies (*p*J) of the two different datasets are determined by integrating the zone I areas of the *q*-*V* pinched hysteresis loops (Fig. S1B). Data show that the triangular wave does not influence stored energy values.

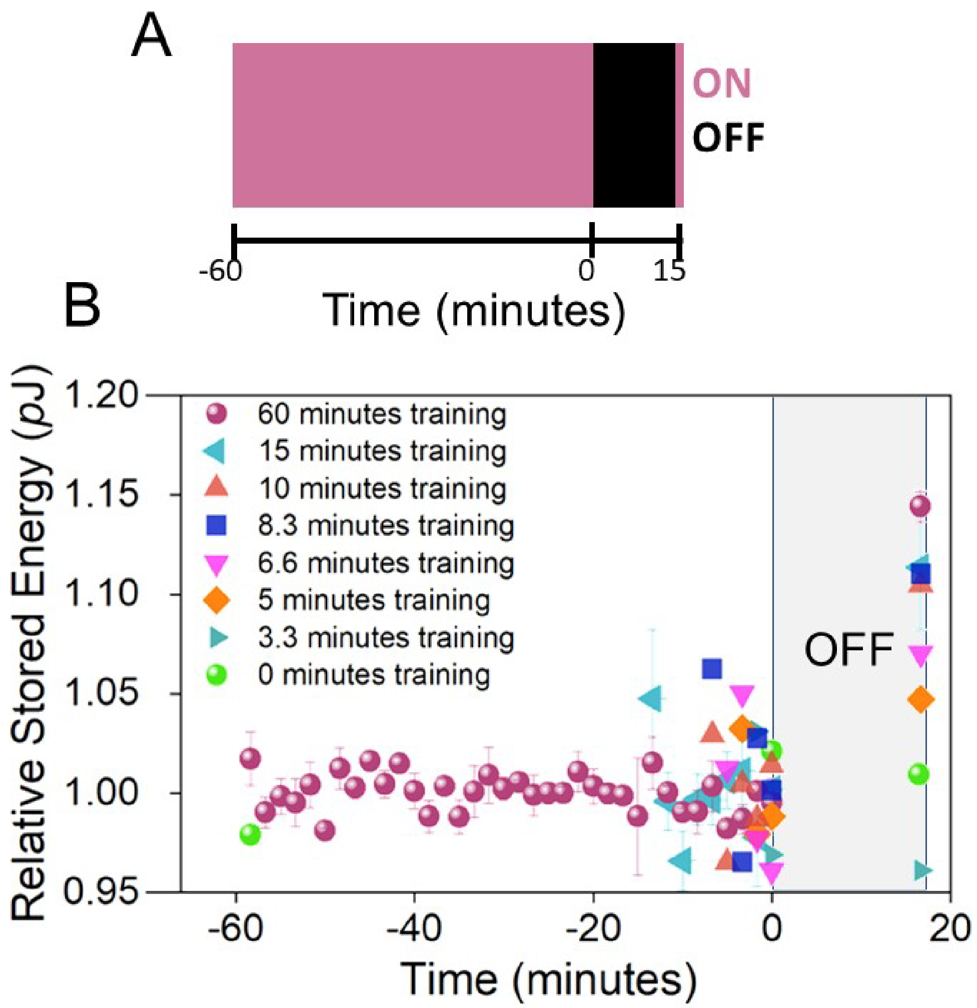

Fig S5. Minimum training period needed for LTP. (A) Graphical representation of when the sinusoidal voltage waveform in both ON (purple stripes) or OFF (black stripe) intervals – comparable to Fig. S2A for the 60 min. training period. The training period is followed by a 15 min. OFF period, which is then followed by a single 99 s sinusoidal voltage waveform. Training periods varied between 60 and 0 min. Only the 60 min. training period is represented, with all additional training periods having a shorter continuous application of the sinusoidal voltage waveform. The end of the training period was set at 0 min. to allow for a proper comparison of data points after the end of the training period. (B) Stored energies (*p*J) obtained by integrating the zone I areas of the q-V pinched hysteresis (Fig. S1B). Training periods were varied in time (0, 3.3, 5, 6.6, 8.3, 10, 15, and 60 min.) to determine the influence of training on stored energy values. In all cases, the training period was followed by a 15 min. OFF period (light gray stripe) and a single 99 s sinusoidal voltage waveform. From these data, we determined the minimum training period required to induce LTP was 8.3 min. (corresponding to 5 sinusoidal voltage waveform cycles).

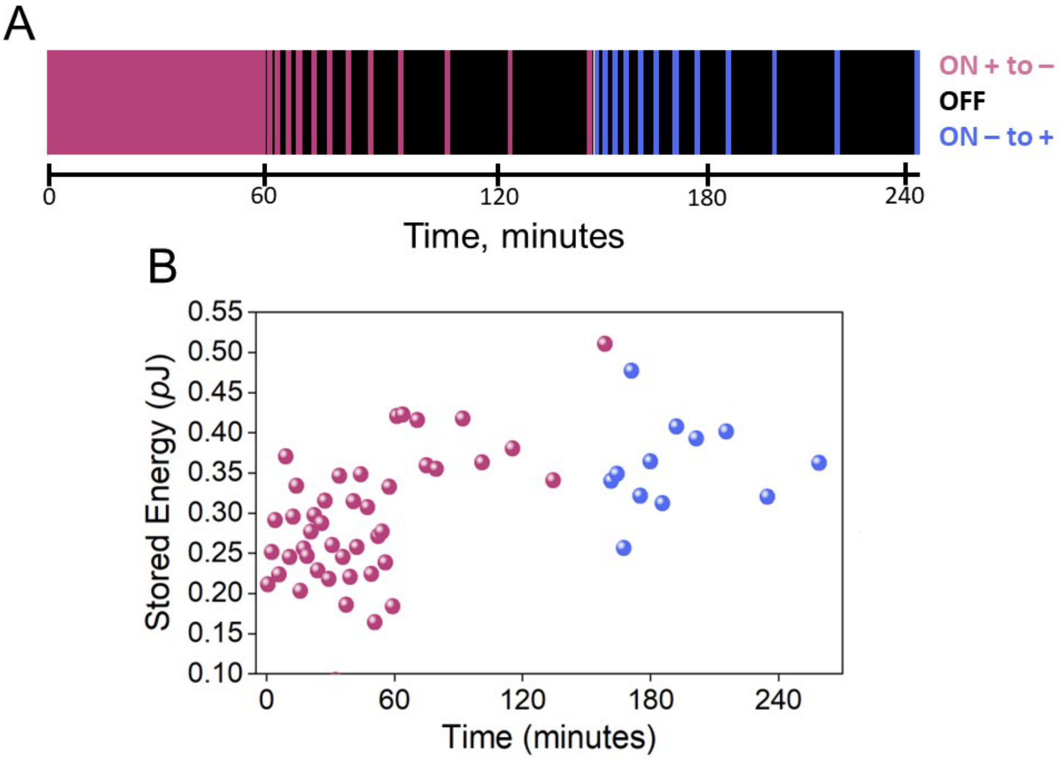

Fig S6. Erasing does not occur when single sinusoidal voltage waveforms are applied. (A) Timeline showing when the sinusoidal voltage is ON (purple stripes) or OFF (black stripes), where ON corresponds to the applied voltage sequence of 0, 0.12, 0, -0.12, and 0 V, in equal steps of 0.97 μV (Fig. S1B, purple arrows), comparable to Fig. S2A. However, after ~160 min., the polarity of the applied sinusoidal voltage waveform was reversed and followed the sequence 0, -0.12, 0, 0.12, and 0 V, in equal steps of 0.97 μV (Fig. S1B, blue arrows). After the training period, the ON + to - and ON - to + are identical to each other. (B) Stored energies (*p*J) determined by integrating the areas of the *q*-*V* pinched hysteresis loops in zone I collected during the ON periods (Fig. S1B). After the initial training period, an increase in the stored energy, comparable to Fig. 2C and Fig. S2B, is observed. Reversing the polarity of the sinusoidal voltage waveform does not affect the stored energy values (blue dots), suggesting that erasing only occurs if the reverse polarity sinusoidal voltage waveform was applied repeatedly, as shown in Fig. 3B.

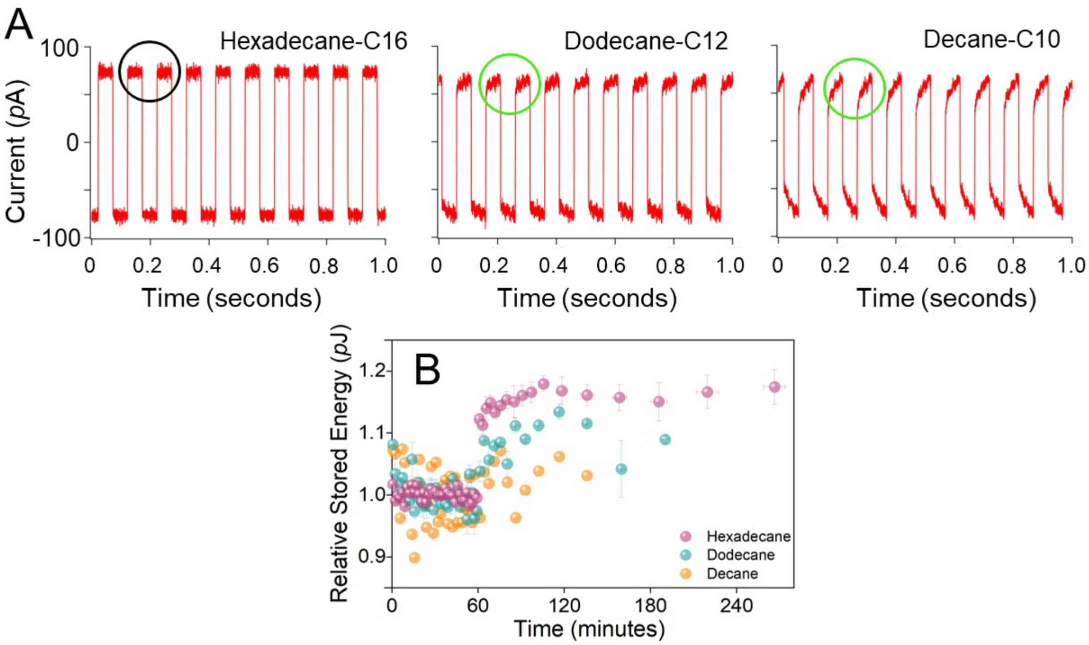

Fig. S7. Membrane permeability influences the development of LTP. (A) Capacitive currents (corresponding to the 10 mV-10 Hz triangular voltage waveform) plotted as a function of time for different chain length oils. The current profiles from the dodecane-based (top, middle) and decane-based (top, right) DIBs deviate from the typical square wave profile (flat top, black circle), indicative of a highly resistive, tightly packed membrane seen in hexadecane-based DIBs (top, left). These deviations (green dots) are good indications of increased ion permeation in less-organized lipid bilayers. (B) Stored energies (*p*J) as functions of time for DIBs in the different alkanes. Decreasing the alkane chain length results in a decrease in stored energy as a function of time – due to increased ion permeability through the bilayer. In the case of decane, ion conductance is high enough that there is no detectable change in stored energy compared to the baseline 60 min. training period data.

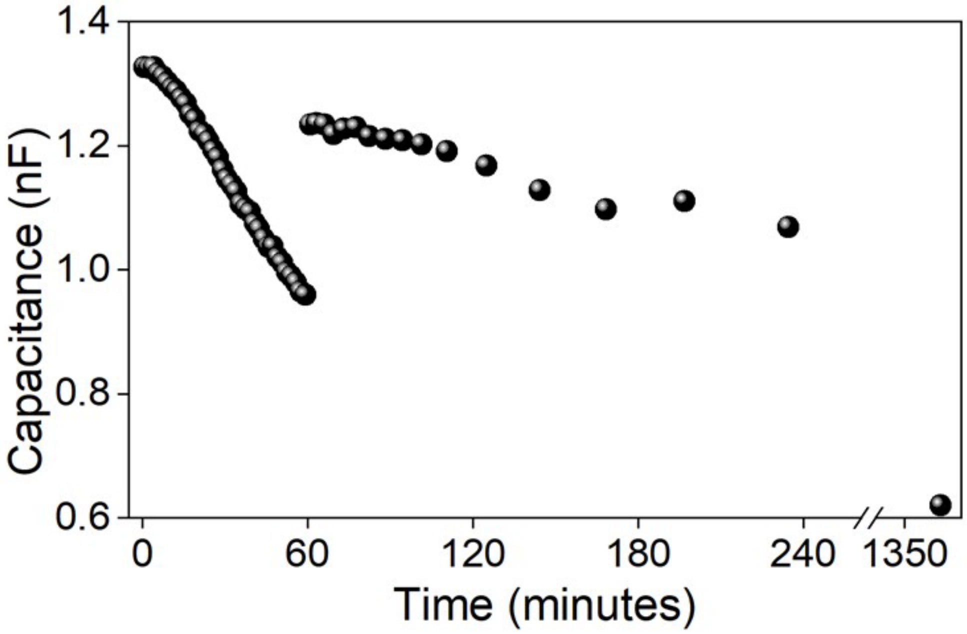

Fig. S8. Initial capacitance at zero volts (C_0) versus time. The slope of C_0 vs. time indicates the rate of entropy increase due to dissipation. Entropy as a result of energy dissipation is a driving force for capacitive memory and the production of LTP in the membrane. The change in the slope of C_0 vs. time, in transitioning from continuous training to isolated, individual capacitive measurements of LTP separated by time intervals, is related to the change in the entropy production rate. Entropy increases at a faster rate during continuous training than after applying gaps in time between individual scans, consistent with the steeper slope seen during training.

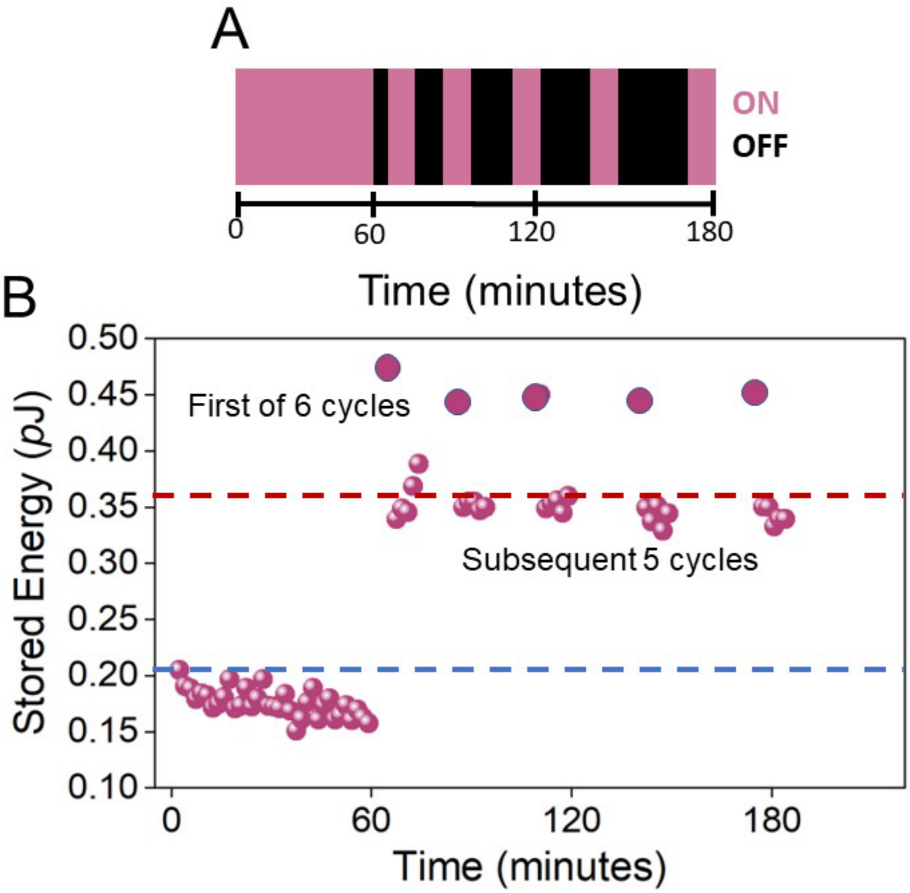

Fig S9. Training and LTP. (A) Graphical representation of ON (purple stripes) or OFF (black stripes) time intervals of the applied sinusoidal voltage waveform. Each ON period after the initial 60 min. training interval consists of 6 cycles of the sinusoidal voltage signal. (B) Stored energies (*p*J) determined by integrating the areas of the *q*-*V* pinched hysteresis loops in zone I (Fig. S1B). At the beginning of each ON interval in the LTP regime (for times after the 60 min. training period), the initial stored energy is highest (large dots), but then relaxes with a characteristic time constant associated with the equivalent RC circuit (smaller dots). The blue and red dashed lines denote the average stored energies during training and LTP, respectively, from the data shown in Fig. S2B. In the LTP region, the stored energy in the first cycle of each 6-cycle ON interval decayed to the average value (red line) by the second cycle, indicative of capacitive relaxation times that were significantly shorter than the time scales for membrane charging and induction of LTP.

Table S1. Statistical parameters from Independent Sample *t*-test. A p-value of ≤ 0.05 indicates statistically meaningful differences.

Figure	Levene's Test p-value	Independent Samples *t*-test p-value
2C	0.272	0.058
2C	0.550	7.11E-33
3D	0.023	1.66E-10
S2B D2O	0.000309	7.39E-50
S2B H2O	0.843	4.07E-73
S2D D2O	0.970	1.09E-61
S2D H2O	0.216	5.06E-45

Table S2. Statistical parameters from Dunnett's two-sided *t*-test. A p-value of ≤ 0.05 indicates statistically meaningful differences.

Figure	**Levene's Test p-value**	**Dunnett's Two-Sided *t*-test p-value**
3B, LTP to Training	0.215	1.56E-8
3B, LTP to Erasing	0.215	1.56E-8

Table S3. Time dependent DIB diameter as a function of time.

Time (Hours)	**Left Drop Diameter (Pixels)**	**Right Drop Diameter (Pixels)**
0	195.0	181.1
16.5	187.0	181.0
24.5	186.5	177.8
42.5	184.1	176.0
65	185.0	177.2

SI References

1. A. Soper, C. Benmore, Quantum differences between heavy and light water. *Phys. Rev. Lett.* **101**, 065502 (2008).
2. W. Zhang, X. Chen, A. C. Van Duin, Isotope effects in water: Differences of structure, dynamics, spectrum, and proton transport between heavy and light water from ReaxFF reactive force field simulations. *J. Phys. Chem. Lett.* **9**, 5445-5452 (2018).
